# A qualitative investigation of the role of the family in structuring young people’s alcohol use

**DOI:** 10.1093/eurpub/ckv123

**Published:** 2015-07-03

**Authors:** Nina Jacob, Georgie J. MacArthur, Matthew Hickman, Rona Campbell

**Affiliations:** 1 Cardiff University School of Social Sciences, Cardiff, UK; 2 School of Social and Community Medicine, University of Bristol, Bristol, UK

## Abstract

**Background:** Few qualitative studies have investigated young people’s perspectives around influences on substance use. We aimed to examine young people’s understandings, attitudes and experiences around alcohol, tobacco and cannabis use and factors influencing substance use behaviour. **Methods:** Qualitative interview study involving 28 young people (13 males and 15 females) aged 18–20 years, recruited purposively on the basis of substance use, who were participating in the Avon Longitudinal Study of Parents and Children. Interviews were conducted at participants’ homes or at local cafés. Audio data were transcribed verbatim, systematically coded and analysed inductively using a constant comparative approach. **Results:** Parental attitudes and behaviours and the nature of communication emerged as critical factors structuring young people’s alcohol use. Initiation of alcohol use was frequently mediated by parents early in adolescence, with the home recounted as a primary site of early drinking experiences. Later in adolescence, young people perceived a more permissive stance towards alcohol use, with broad acceptance of high levels of consumption and recognition of drinking as a cultural norm during adolescence. In contrast, young people reported a more prohibitive and discouraging stance from their parents towards tobacco and cannabis use, and the use of these substances appeared to be of greater parental concern. **Conclusions:** Interventions involving parents or guardians have a critical role to play in the prevention of harms arising from alcohol use during adolescence. However, such interventions are needed in conjunction with individual, school, community and environmental interventions to shift cultural norms across the population and to facilitate effective prevention.

## Introduction

Risk behaviours such as alcohol, tobacco and drug use, commonly emerge during adolescence and are associated with a range of harms including injury, violence, sexual risk behaviour, poor academic achievement, mental illness and subsequent dependence. Continuation of risk behaviours into adulthood can further impact on morbidity and mortality later in life,^1^^–^^4^ thus prevention of such behaviour in young people is a major public health goal.^2^

In Europe, an average of 87% of 15–16-year olds have drunk alcohol at least once, and in the United Kingdom, the prevalence of alcohol and drug use is high compared with other European countries.^5^ One-quarter of young people aged 15–16 years have used cannabis in the United Kingdom^5^ and one-third were recently reported to be harmful or hazardous drinkers.^6^ Alcohol use is of particular concern, with the United Kingdom ranked 3rd of 36 countries in the proportion of young people being drunk in the past month.^5^

A number of factors influence the extent and pattern of alcohol and substance use, including genetic, family and peer influences and wider social, environmental and legislative contexts,^7^^,^^8^ While peers and social norms have a particular prominence in shaping behaviour during adolescence,^9^^–^^12^ the family environment plays a major role in influencing patterns of alcohol use, both prior to and during adolescence. More specifically, family influences such as parental monitoring, family closeness, frequent communication and parental disapproval have been reported to be protective, delaying initiation to alcohol use, frequency of alcohol use, binge drinking, drunkenness,^13^^,^^14^ substance use and risky sexual behaviour.^15^ In contrast, family conflict, poor communication, parental drinking and parental permissiveness increase the risk of alcohol and substance use among young people.^16^^–^^18^

Although quantitative studies have demonstrated the importance of family characteristics, comparatively few qualitative studies have explored the views and perspectives of young people and the role of parents or guardians in relation to alcohol use behaviour. To date, qualitative studies demonstrate that parents consider experimentation with heavy alcohol use to be somewhat inevitable and thus primarily report promotion of safe and sensible drinking, while aiming to maintain good quality, open relationships with their children.^19^^–^^23^

In this article, we report findings of a qualitative study conducted with young people participating in the Avon Longitudinal Study of Parents and Children (ALSPAC) in Bristol, England, which aimed to explore young people’s understandings, attitudes and experiences around alcohol, tobacco and cannabis from early through to late adolescence. In particular, we report findings relating to the role of the family in structuring young people’s incipient relationship with alcohol.

## Methods

### Sampling

ALSPAC is a longitudinal population-based cohort study of children born to mothers resident in the former Avon region in the South West of England who had expected delivery dates between 1 April 1991 and 31 December 1992.^24^ The current analyses are based on a nested qualitative exploration of participants’ views and experiences of alcohol, tobacco and cannabis. The study adopted purposive sampling to speak with young people with a variety of substance use behaviours. Between April 2009 and September 2010, ALSPAC participants responded to a questionnaire which included questions relating to their alcohol, tobacco and cannabis use (for instance age of first drink; frequency of alcohol use; and cannabis and tobacco use in the previous 6 months). Potential participants (who had reported that they were willing to be contacted for further studies) were then identified on the basis of responses to these questions. In total, 147 invites were sent out and a total of 28 young people aged between 18 and 20 years participated in the study (Supplementary Table S1). Informed consent was obtained from participants, and ethical approval for the study was granted from the ALSPAC Ethics and Law Committee and the Local Research Ethics Committees. Please note that the ALSPAC study website contains details of all the data that is available through a fully searchable data dictionary.^25^

### Data collection

In-depth interviews (conducted by N.J.) facilitated by a topic guide, which explored participants’ views and experiences on substance use, were the principle means of data collection. These were mainly conducted in the community at participants’ homes, universities or local cafés. The interviews focussed on their personal history of alcohol, tobacco and cannabis use; drug and alcohol education; harms and risks; social networks and environments associated with use; attitudes, beliefs, intentions and aspirations around substance use and resistance to use. Specific questions relating to family reactions to substance use were asked, although their role in such behaviours was discussed in relation to the broad themes covered. Interviews lasted between 30 minutes and 2 hours were digitally recorded and transcribed verbatim. Participants provided signed informed consent and received a £10 voucher for taking part. All interview data were fully anonymized, with participant names being changed to preserve confidentiality.

### Data analysis

Data analysis was ongoing and iterative, with analyses of early data indicating new lines of inquiry to be pursued in subsequent interviews. A constant comparison method was the principle means of analysing the data. First, transcribed data were read and re-read, and emerging themes, categories or concepts were discussed between the study team. The transcripts were coded by N.J. focussing on uncovering participants’ social world and the meanings they attach to different substance use behaviours. These codes were generated to initially break down, examine, compare and begin to categorize the data. These categories were then used to organize the data through a cross-sectional, code and retrieve approach applied across the whole data set using NVivo computer software. In the second phase of the analysis, a constant comparative approach was used to systematically map observations from the data.^26^ This mapping enabled full engagement with the data, constantly refining the analysis, comparing for similarities and differences in the accounts of the respondents, so that all data were carefully considered and categories emerged until the data reached saturation point. Finally, elements of the framework approach^27^ were also developed in the analysis. While not used in its purest form, once explanations from the data began emerging, this approach was utilized, principally its method of charting and tabulating raw data to display patterns and relationships, classifications and typologies.

## Results

### Introducing children to alcohol and the journey through adolescence

The central role of the family in young people’s early experiences with alcohol emerged as a clear theme in this study. For many young people, their first contact with alcohol was in the home, being offered to them by their parents in their early teens. Early introductions to alcohol were moderated by parents where there was an apparent focus on sensible drinking. Young people reported that introductions to alcohol were often in the context of special occasions, such as birthdays or New Year, an approach potentially laying the foundations for an association between alcohol and celebration or enjoyment (Box 1, quotes 1 and 2).

Box 1 Introducing children to alcohol and the journey through adolescence

Quote 1

Obviously the only times I’d really drink with my parents would be New Year and things because obviously they drink with you don’t they on New Year’s. Because leading up to before I was fourteen we didn’t really drink. If it was New Year we’d have the little Belgium lagers which weren’t that bad so they let us get on with it. They’d give us this much and say ‘that’s all you’ll have between you’. Bearing in mind we’d always go round to family friend’s house so there were six kids. They were like ‘you’ve got 24 bottles between the six of you’. (ID 3, F, aged 18)

Quote 2

Thinking about your family, you said that pretty much all of your drinking is centred around the family, so what have their attitudes been towards drinking? When you were first starting, how did they introduce you to it?

R^a^: It was generally just a small glass of wine on my birthday or New Year or Christmas or something.
I^a^: What kind of time would that start happening?
R: With a meal or something so it’s not just have the alcohol on its own. (ID 9, M, aged 19)

Quote 3

I: So when you think back to those times when you first started having alcohol with your friends, what would that involve, how would you get hold of …?
R: I think it was at parties that I’d have at home. Mum and dad would buy it and we’d all have some and hopefully it wouldn’t have had too much, but because we were young we wouldn’t get drunk too quickly and everything.
I: So parents would monitor how much you were having?
R: Yeah they would have been there and like, making sure we didn’t have too much.
I: And would you have got any more or would it just be exactly what they bought for you?
R: Well no we wouldn’t have got any more. We would have just had what they bought and we would have asked them to go out and get more but they probably wouldn’t have said yes. (ID 7, F, aged 19)

Quote 4

I: Do you know how you got it?
R: … When I got a bit older maybe sixteen or seventeen, my Mum used to. She knew I was going to a friend’s so she’d buy me a bottle of wine or something knowing where I was. She’s always been quite good with that sort of thing and I’ve normally not abused it too much.
I: As long as she knows where you are going I guess?
R: Exactly yeah, but she wouldn’t do it often; it wouldn’t do it every single week she’d buy it for me. It might be oh I’m going to go out this weekend could you pick me up a bottle, some cans of cider or something and she’d normally say yes but if I asked every week she would say no so she didn’t just give it to me.
I: So she managed it a little bit then?
R: Yeah. (ID 10, F, aged 19)

Quote 5

I: So it was moderated, so the parents allowed you to have it?
R: The parents allowed us to have it, and you’ll find a lot with all times I’ve been drinking the parents allowed people to have it,… the thing is my parents are always ok with it to an extent but I did what a lot of people did, I would say to my parents ok can I have one can of cider, two cans of cider, and they would be like, ok and actually I would have three or four. (ID 8, M, aged 18)

Quote 6

I felt a little bit restricted, the fact that I couldn’t have so much fun, obviously because you’re that age you’re like ‘oh yeah we’re drinking, we’re really cool. We’re going to do it, we’re going to be awesome’ and then having your parent saying ‘oh you’re only allowed this much’ it’s a bit like ‘oh what, you’re going to rain on my parade but I look back at it now and I think they were only trying to help us because obviously they know the effects of it so I appreciate they’ve helped us out with that effect but then when I got to eighteen I was like ‘yeah I can finally drink as much as I like’ which didn’t work very well. (ID 3, F, aged 18)

Quote 7

Other people had older friends who were getting it for them. One of my friends she used to get it from some people who were older but they were a bit creepy to be fair. I don’t really understand it. I think they were only friends with them because they did that. (ID 22, F, aged 19)

a: R, respondent; I, interviewer.

These initial encounters within the family context were extended once participants entered adolescence, with many describing drinking with friends in the home environment under parental supervision, something that typically occurred between the ages of 14 and 17 years (Box 1, quotes 1 and 3). Parents were reported to provide alcohol but to impose specific limits on the volume consumed in an attempt to moderate consumption, often against the wishes of their children (Box 1, quotes 3 and 4). Many participants adhered to this approach, but some reported exceeding these limits and lying to their parents (Box 1, quote 5) or significantly increasing their consumption once away from the watch of their parents (Box 1, quote 6), often via the provision of alcohol by other friends, siblings or strangers (Box 1, quote 7).

### The perceived permissive approach of parents

Communication with parents was often reported as becoming more open later in adolescence and young people perceived that their parents’ exhibited a permissive stance towards their alcohol drinking, typically once they were legally allowed to drink. Parents were reported to tolerate drunkenness and its effects, displaying an understanding of such behaviour and a view that their children would learn their own lessons around drinking and drunkenness (Box 2, quotes 1 and 2). Young people perceived the threshold for boundary setting or intervention by parents to be high and linked to observable displays of drunkenness, safety precautions and/or more direct impacts on parents, rather than drinking in adolescence *per se* (Box 2, quotes 3 and 4).

Box 2 The perceived permissive approach of parents

Quote 1

I: Have they [*your parents*] said it to you?
R: Yeah they say they don’t particularly want me to go out and get rat arsed but they’ve seen me with a hangover the night after they know that I got pissed. They’ve seen me come down with sun glasses and down a whole thing of coffee… They are perfectly happy to buy me booze, they just prefer if I keep it in moderation but they understand university, the whole scene. They understand that things are going to happen. (ID 1, M, aged 18)

Quote 2

R: Alcohol, she has just said you’ll make your own mistakes and I think once she asked me the next morning, because she heard me being sick and she didn’t really tell me off, just said that wasn’t very clever was it. I think my boyfriend had stayed round that night and she was like, was that you or K? I was like, that was me and she said that wasn’t very attractive was it.

I: So letting you learn from your experiences really then?
R: I think so…. I think she thinks it will go on whether or not she lets me do it, so maybe she finds it easier just to be a bit, well I’ll keep an eye but I won’t get too involved unless I need to. My dad said the same thing. (ID 10, F, aged 19)

Quote 3

That’s the impression I get, amongst my friends, if you are going out and not getting absolutely off your face and do it responsibly then your parents leave you to it but if you’re going out and coming back absolutely plastered then they will be like, ‘You’re not drinking anymore’. (ID 22, F, aged 19)

Quote 4

Drinking, she’s [*my mother’s*] ok with as long as I’m responsible. She says ‘as long as you know what you’re doing and you don’t push yourself over your limit and you don’t wake me up when you get home that’s fine.’ That’s all she worries about, if I hurt myself or if I come in and trash the house and wake her up. (ID 3, F, aged 18)

Quote 5

I: How much would you say that you normally drink when you do go out?
R: Maybe like three glasses of wine, three ciders and a couple of shots, which isn’t very much really, not like most people. (ID 7, F, aged 19)

Quote 6

I: And why will you get very drunk?
R:…It will take quite a long time to get there so I will keep drinking more and more and then suddenly it will hit me and I will be really drunk. But I don’t really get drunk to the point where I don’t know like what I’m doing and can’t sort of control myself anymore, I don’t do that. (ID 28, F, aged 20)

Quote 7

I: Going back to your parents – what are their general attitudes towards drinking?
R: I think they don’t mind it as long as you don’t get stupid with it. (ID 22, F, aged 19)

Despite these perceptions, participants were aware that their parents would prefer them not to drink to excess, yet this did not seem to alter behaviour. Participants often highlighted that their drinking behaviour was limited or controlled, thus being in keeping with their parents’ preferences. However, a substantial proportion frequently consumed greater than recommended daily limits when drinking (e.g. Box 2, quotes 5 and 6 and Supplementary Table S1), thus demonstrating a lack of awareness around the amounts of alcohol that would constitute safe and sensible drinking.

In part, this may be linked to the lack of evidence given, since parents did not appear to provide unambiguous and specific guidance to their children on what the boundaries were when it came to alcohol. While it was clear from young people that their parents would prefer them to be sensible, little information was conferred about the amounts that should be consumed if drinking sensibly (Box 2, quotes 3 and 7). Similarly, there was little evidence from participants that their parents discussed the negative consequences of alcohol use, particularly the longer-term harms associated with drinking.

### Modelling of parental behaviour

The role of parents in introducing their children to alcohol, and the perceived permissive approach of parents, highlighted the role of the family as a major contributor to the broad ‘accepting’ culture around alcohol use that is currently evident in the United Kingdom (Box 3, quote 1). Participants’ reflections of their parents’ own use of alcohol, albeit moderate for most, highlighted the extent to which alcohol drinking was seen as a normal aspect of family life (Box 3, quote 2). In some cases, young people’s discussions about their parents’ drinking behaviour were interpreted as sanctioning the participants’ alcohol use. Thus, there were times when parents’ attempts to restrict alcohol use did not necessarily have a positive impact on their children’s behaviour (Box 3, quotes 3 and 4).

Box 3 Modelling of parental behaviour

Quote 1

You’ve got to be eighteen, but it’s not against the law, it’s not particularly frowned upon because your parents do it, and ok they never get drunk, but it’s not something that people would be like, oh my God that’s terrible. (ID 10, F, aged 19)

Quote 2

My parents have a drink every evening mostly, two at the most which is a glass of wine but again that’s with food so that’s not just by itself. It’s not really anything that has been a big deal to them at all. (ID 19, M, aged 19)

Quote 3

Yeah they do accept it I mean because everyone’s done it, that’s what they say. I mean I’ve been at home when my dad’s been puking because he’s been drinking and he’s what, fifty three, fifty four now. I can remember we’ve left a family barbeque thing and my dad’s been opening the car door to be sick. Even if I was sick I’ve got ammo against him to defend myself. (ID 8, M, aged 18)

Quote 4

I: So your parents do have a different attitude towards alcohol than to smoking and cannabis yeah?
R: Yeah they do because it would be a bit hypocritical for them to tell me to not drink because they do. (ID 1, M, aged 18)

Quote 5

I: But do you know their [*your parents’*] thoughts and feelings about it?
R: Umm I suppose fairly similar-ish to mine in terms of going and having and having a drink. They don’t go out and get absolutely drunk themselves. They have done in the past when they were sort of my age or something but nowadays it doesn’t happen. I’ve never seen them drunk. (ID 9, M, aged 19)

Quote 6

R: My parents don’t drink either
I: Do they not?
R: Wine at Christmas or when they have friends round but they don’t drink either. Some people’s parents have a bottle of wine a night. My parents don’t. So I suppose if your parents drink it could potentially influence you to from a younger age. (ID 17, F, 19)

Quote 7

R: My Granddad was an alcoholic and now he’s got dementia because of alcoholic processes killing his brain cells. And my Dad does drink every night, a lot a lot a lot, yeah so.
I: And what do you think about that?
R: I think it’s stupid yeah. I just don’t see the point in drinking absolutely loads.
I: Do you think that’s had an effect on you? Your Granddad or your Dad?
R: Umm yeah maybe I don’t want to drink because they drunk lots. That might be something, probably. (ID 7, F, aged 19)

Quote 8

R: My dad he’s like an alcoholic, he’s got a drink problem. He says he hasn’t, but he’s in denial, like he has. And I seen him drunk like most of my life. And I always thought to myself, ‘ah no I’m never gonna drink’. But..
I: So how did you go from that thought[
R:]I don’t know. I just like grew out of that little stage and it’s like I didn’t think I’d get to how I am now. It’s just like.. It’s just, it’s just bad. (ID 16, F, aged 18)

Notably, however, for a minority of individuals, modelling of their parents’ drinking habits was evidenced in a more moderate or abstemious approach to alcohol. These young people highlighted their parents’ lack of drinking or drunkenness and likened their view or behaviour to that of their parents (Box 3, quotes 5 and 6). For a small number of additional participants, experiences of alcoholism within their family brought home the longer-term harms of alcohol use and the possibility of dependence, factors that were not discussed in other families. Such observations divided these young people to those reporting greater care in their drinking patterns (Box 3, quote 7) and those for whom alcohol was similarly a central or significant part of their life (Box 3, quote 8).

### Contrasting parental attitudes towards tobacco and cannabis use

#### Prohibitive approach

In contrast to the perceived tolerance of parents towards alcohol consumption, young people reported a more prohibitive approach of their parents towards tobacco and cannabis use (Box 4.1, quotes 1 and 2). This was particularly the case for tobacco, where parents were clear about their negative attitudes towards smoking and appeared to be more likely to provide information about the long-term consequences of tobacco use (Box 4.1, quote 3). Direct conversations about cannabis appeared to be less frequent, with many participants making assumptions about their parents’ negative views (Box 4.1, quote 4). At times, this was thought to be linked to parents’ own lack of experience with cannabis or an assumption that their children had not come into contact with it (Box 4.1, quote 5). Nevertheless, at times, young people spoke of the severe consequences that would result if their parents became aware that they had used cannabis (Box 4.1, quotes 6 and 7), indicative of participants’ interpretation that their parents held firm views and had clear rules and boundaries.

Box 4 Contrasting parental attitudes towards tobacco and cannabis use

4.1. Prohibitive approach

Quote 1

I: You said that your parents are or were ok with you drinking one or two cans when you were younger. What are their general attitudes towards these three substances that we are talking about?

R: What with me now or just in general?
I: In general what would you say?…
R:… Umm, attitudes to weed don’t do it, attitudes to smoking, don’t do it, alcohol, do it as long as you are safe. That’s kind of their opinion now (ID 8, M, aged 18)

Quote 2

Mum’s a doctor so she would kill me if I started smoking and I’ve got asthma so it would really screw me up. Umm, Dad used to smoke but would still kill me if I started smoking and it would be the same with cannabis really. They are not that strict but they would kill me if I started smoking cannabis. But with booze they don’t mind, they understand that I’m going to get pissed (ID 1, M, aged 18)

Quote 3

So if you think about alcohol, tobacco, cannabis, where do you think you first got information about those things from?

R: Umm my parents.
I: Was it?
R: Yeah. My mum and dad both smoked cigarettes, and then they both quit, so they kind of gave me all the, well my mum is a nurse so she gave me the what’s wrong with it all, why you shouldn’t do it, and how it’s really not that great… (ID 4, M, aged 15)

Quote 4

I: What do you think, or do you know what your parents’ attitudes are towards smoking, drinking and cannabis? Have you ever had a conversation about it in the family?
R: Umm obviously they don’t like me getting absolutely wasted but they don’t mind me drinking. If I am normal about it, if I am sensible about it I guess. As for smoking cigarettes, they don’t like it. My sister smokes and they don’t like it. Cannabis, I have never really had the conversation with them. I think it is probably the same as smoking, they think it is unnecessary probably. (ID 11, F, aged 19)

Quote 5

They [*my parents*] have never said anything to either me or my sister about drugs. I have heard my Mum say to family on the phone that she doesn’t think we are around it at all… Maybe she sees it as whatever she used to do when she was younger; she assumes that we’ll try too. Maybe she hasn’t mentioned any drugs to us because that was never something she did, I don’t know. She has never actually said. The only one I have had ‘don’t do it’ is smoking and I agree with that. (ID 10, F, aged 19)

Quote 6

R: My Mum is completely against drugs. She despises them, she told me she hates them and I haven’t even told her I tried it because I knew she would go mental because obviously I was there when she found out that my brother was doing it and the reaction she had, I was like ‘yeah I better not tell her’ purely for the fact that she went berserk and said you’re messing your life up (ID 3, F, aged 18)

Quote 7

I: So you have never really had specific conversations in the family about it then, specifically about your use as well? They haven’t sat down and said never have cannabis because it is not [

R: [I know that if I ever did I would be straight out of the house, I know that for a start (ID 19, M, aged 19)

4.2. Discouraging approach

Quote 1

I: And what about the other two?
R: Umm my Mum used to smoke but she gave up when she had me and my brother. I never have and I just said no I never have. They are happy that I don’t but I think if I did they would try and encourage me to stop but they would be pretty easy. Weed, I’m not really sure about weed, I think they wouldn’t go nuts about it, they would probably prefer I didn’t.
I: But they haven’t actually ever said to you look, don’t do this?
R: No
I: So they accept it how it is?
R: Yeah they probably don’t know about weed. I probably keep that quiet from them but I don’t get the feeling they’d fly off the handle about it. (ID 12, F, aged 19)

Quote 2

I: Have they ever had conversations with you about anything like that?
R: Umm yeah I mean they’ve said at the end of the day it’s like free choice, free will, but my Mum and Dad told me the dangers and they said ‘look this is what could happen to you, cannabis might provide you with a good relief at first but then the day after or a couple of hours after it just messes with you’. They’ve never actually said don’t do it they’ve just said this is what will happen. (ID 5, M, aged 18)

Quote 3

R: She [*my mum*] just got really annoyed, well not annoyed she got hurt I guess cos I was being angry and defensive, you know, I’m smoking weed, I didn’t want to tell her.
I: Yeah, yeah.
R: And um, she must have just pieced it together, I guess and then, um, it just didn’t stop. I kept telling her that I wasn’t but I had and that I’d stopped now and things like that. I just grew to become better at hiding it… I think she did sort of know that there was a few lies coming out, but how many…
I: So there’s never been like a proper open conversation about it really then?
R: No, there has um.. Now there is…
I: So you’re quite open about it now then really?
R: Yeah. (ID 23, M, aged 19)

Quote 4

I: I’ve been asked about it before and I said I’ve done it [*cannabis*] and she [*my mum*] said don’t get involved.
I: Mum has said this to you?
R: Yeah
I: So she just asked you if you had?
R: Pretty much. This was at a house party, a group of my friends have quite big weekends basically umm so there was a load of them and Mum was like ‘did you smoke any weed?’ and I was like ‘well a bit’ and she was like ‘I don’t want you touching stronger substances’ and I wouldn’t. (ID 6, M, aged 18)

Quote 5

I: So have you had any conversations in the family about it?
R: I have in the past.
I: But nothing saying that you can’t do it?
R: It was initially and then they sort of gave in.
I: So are you quite open with them or is it something that’s not really spoken about?
R: Well I’m open with them now, but I didn’t used to be.
I: So you do have conversations with them about it?
R: Yeah.
I: And they accept that you’re going to be smoking weed and taking pills and things?
R: They accept it, they’d rather I didn’t of course, but they accept it. (ID 27, M, aged 19)

Quote 6

R: And then she caught me [*smoking*] a couple of times.
I: So what was that like then?
R: She was like ‘oh if you’re gonna do it’, well no first it was like ‘no you can’t smoke’ but I was sort of like ‘well I can do whatever I like’, so then she was like, ‘well not in the house’.
I: So she’s kinda reluctantly accepting that it happens then.
R: Yeah, yeah. (ID 28, F, aged 20)

#### Discouraging approach

A number of families took a more discouraging rather than prohibitive approach to substance use. Young people spoke of their parents’ tolerance of their pattern of substance use (Box 4.2, quote 1) but continued to focus on the risks associated with substance use, as evidenced above (Box 4.2, quote 2). Although participants hid their substance use from parents earlier in adolescence, this more tolerant approach of parents seemed to encourage more open communication by the young people as they moved through adolescence (Box 4.2, quote 3). Participants reported that parents displayed a reluctant acceptance of their substance use within certain boundaries, often in response to young people’s continued use of the substance in question (Box 4.2, quotes 4 and 5). Such parental acceptance frequently followed a reportedly firm stance, which waned as young people tested or directly resisted boundaries, such that parents made attempts to find an acceptable middle ground (Box 4.2, quote 6).

## Discussion

Our study uniquely explored young people’s perspectives around alcohol, tobacco and cannabis use and their interpretations of parental attitudes towards substance use. Family influences on alcohol use and the contrast between communication around alcohol and other substances emerged as major themes.

First, the family emerged as a key contributor to the accepting culture around adolescent alcohol use. Participants reported that their parents introduced them to alcohol early in adolescence, provided them with alcohol in these early years and subsequently took a comparatively tolerant approach towards drinking later in adolescence. Boundaries frequently appeared to be set around displays of drunkenness and irresponsible behaviour, and there was little focus on the longer-term consequences of alcohol drinking. Second, and in contrast to the perceived approach of parents towards alcohol use, many participants described their parents’ firm stance against tobacco and drugs, and their greater focus on the long-term implications of such behaviour. Young people highlighted that parents imposed clearer boundaries. While some parents reportedly came to accept such behaviour later in adolescence, this tended to follow an initial disapproval or resistance to use of the substance in question.

Our findings are echoed in qualitative studies with parents conducted in the United Kingdom and Australia. These studies have reported that parents aim to have a positive, trusting and communicative relationship with their children but feel a resignation or powerlessness around their alcohol consumption, both of which lead primarily to adoption of a harm reduction approach.^19^^–^^21^ Parents have also been reported to encourage moderation and to normalize alcohol use, either owing to a view that drinking is a normal part of life^20^^,^^22^ or to avoid a prohibitive stance in case this inadvertently encourages their children to drink greater volumes through rebellion.^28^ Such parental behaviour may stem from views that peers or the wider British culture have a comparatively greater influence, leading to an underestimation of parental impact^20^^,^^28^; a pressure felt by parents to act a certain way based on other parents’ behaviour^19^ or a view that social norms are such that their children should be allowed to drink to prevent them from feeling socially isolated.^19^

This accepting approach of parents towards adolescent alcohol use is at odds with guidance from the Chief Medical Officer for England, which states that young people should not drink until the age of 15 years, should not exceed adult daily limits and if they do drink, should do so in moderation and under parental supervision.^8^ Early experiences of alcohol consumption were frequently at home under supervision but were prior to the age of 15 years for some young people, and parents were aware that young people were drinking over recommended limits later in adolescence. Interestingly, studies report that parents often feel ill-equipped to discuss issues around alcohol use with their children,^20^ suggesting that specific guidance for parents may be needed to reduce inadvertent harms.

In agreement with other studies, and in contrast to parents’ perceived approach towards alcohol, we identified a more prohibitive stance of parents relating to tobacco and drug use. For these substances, parents have been reported to be more focused on preventing or dissuading use, whereas for alcohol, the focus is on encouraging their children to drink in a certain way,^23^ a more nuanced message to convey. Similarly, other studies have reported that parents view drugs and tobacco as unequivocally deleterious to health and consider prevention of use of these substances a greater priority compared with alcohol.^19^^,^^21^^,^^22^ In reflection of these findings, young people in our study frequently displayed a lack of awareness of the longer-term health consequences of drinking but acknowledged the potential impacts of smoking tobacco and cannabis.

While peers have been reported to play an important part in determining patterns of substance use during adolescence,^11^^,^^29^^–^^31^ our study highlights the importance of the home environment and parenting style in shaping young people’s attitudes towards alcohol and other substances. Our findings, and those of others,^14^^,^^17^^,^^18^^,^^32^^,^^33^ suggest that there is considerable scope for further research to optimize family interventions and to harness the role of the family in shaping young people’s alcohol use behaviour. In this way, our findings highlight the importance of enhancing awareness among parents of their influence on their children’s alcohol use, both in terms of attitudes towards adolescent drinking and their own behaviour. In addition, they demonstrate the need for greater familiarity with the guidance around young people and safe alcohol use,^8^ as well as wider information about the short- and longer-term impacts of drinking. To date, evidence demonstrates small but consistent and persistent beneficial effects of family-based interventions over the medium- and longer-term^34^^,^^35^ and studies report a greater benefit of interventions that target parents together with young people compared with either group alone.^36^^,^^37^

There are several limitations to our study, which must be noted. As with all qualitative research, the generalizability of these findings are limited to the young people in this study, although a diverse group of young people were sampled with a range of different substance use behaviours and socio-economic positions, and gender was balanced among participants. Young people’s self-reported alcohol behaviour can also be different to what they are actually drinking, either via under- or over-reporting, although the detail provided in individual interviews would suggest that this was not the case for the majority of participants in our study. Finally, we did not triangulate our findings by exploring the views of parents. However, we consider our focus on young people’s interpretations of their parents’ attitudes, behaviours and discussions to be a strength of the study, since this is likely to be a major influencing factor in the development of their intentions relating to substance use.

## Conclusions

Our findings illustrate the role of the family as a contributor to the tolerant culture around adolescent alcohol use in the United Kingdom and the implications of alcohol as a social norm in the population. Our study also provides evidence in support of family-based preventive interventions but suggests that education, guidance and intervention are needed across the social, environmental and legislative context to shift cultural norms and to effectively facilitate a change in behaviour in the young adult population.

## Supplementary data

Supplementary data are available at *EURPUB* online.
